# Novel haplotypes and networks of *AVR-Pik* alleles in *Magnaporthe oryzae*

**DOI:** 10.1186/s12870-019-1817-8

**Published:** 2019-05-16

**Authors:** Jinbin Li, Qun Wang, Chengyun Li, Yunqing Bi, Xue Fu, Raoquan Wang

**Affiliations:** 1 Agricultural Environment and Resources Research Institute, Yunnan Academy of Agricultural Sciences, Kunming, China; 2 The Ministry of Education Key Laboratory for Agricultural Biodiversity and Pest Management, Yunnan Agricultural University, Kunming, China

**Keywords:** *Magnaporthe oryzae*, Effector, *AVR-Pik*, Evolution

## Abstract

**Background:**

Rice blast disease is one of the most destructive fungal disease of rice worldwide. The avirulence (*AVR*) genes of *Magnaporthe oryzae* are recognized by the cognate resistance (*R*) genes of rice and trigger race-specific resistance. The variation in *AVR* is one of the major drivers of new races. Detecting the variation in the *AVR* gene in isolates from a population of *Magnaporthe oryzae* collected from rice production fields will aid in evaluating the effectiveness of *R* genes in rice production areas. The *Pik* gene contains 5 *R* alleles (*Pik*, *Pikh*, *Pikp*, *Pikm* and *Piks*) corresponding to the *AVR* alleles (*AVR-Pik/kh/kp/km/ks*) of *M. oryzae*. The *Pik* gene specifically recognizes and prevents infections by isolates of *M. oryzae* that contain *AVR-Pik*. The molecular variation in *AVR*-*Pik* alleles of *M. oryzae* and *Pik* alleles of rice remains unclear.

**Results:**

We studied the possible evolutionary pathways of *AVR-Pik* alleles by analyzing their DNA sequence variation and assaying their avirulence to the cognate *Pik* alleles of resistance genes under field conditions in China. The results of PCR products from genomic DNA showed that 278 of the 366 isolates of *M. oryzae* collected from Yunnan Province, China, carried *AVR-Pik* alleles. Among the isolates from six regions of Yunnan, 66.7–90.3% carried *AVR-Pik* alleles. Moreover, 10 *AVR-Pik* haplotypes encoding five novel *AVR-Pik* variants were identified among 201 isolates. The *AVR-Pik* alleles evolved to virulent from avirulent forms via stepwise base substitution. These findings demonstrate that *AVR-Pik* alleles are under positive selection and that mutations are responsible for defeating race-specific resistant *Pik* alleles in nature.

**Conclusions:**

We demonstrated the polymorphism and distribution of *AVR*-*Pik* alleles in Yunnan Province, China. By pathogenicity assays used to detect the function of the different haplotypes of *AVR*-*Pik*, for the first time, we showed the avoidance and stepwise evolution of *AVR*-*Pik* alleles in rice production areas of Yunnan. The functional *AVR-Pik* possesses diversified sequence structures and is under positive selection in nature.

**Electronic supplementary material:**

The online version of this article (10.1186/s12870-019-1817-8) contains supplementary material, which is available to authorized users.

## Background

In the long history of parasitism, adaptive mutations have occurred between hosts and pathogens, and selection pressure has traditionally been considered the main force driving this coevolution. To date, two hypotheses have been proposed regarding these dynamics: arms race and trench warfare evolution between host resistance genes (*R*) and pathogen avirulence genes (*AVR*) [1]. The arms race hypothesis is considered the principal hypothesis, in which both host *R* genes and pathogen *AVR* genes are under directional selection and the alleles are derived by mutation. In brief, pathogens evolve a virulence gene to overcome host defense, while the hosts evolve a new resistance allele to defeat the virulence genes of the pathogen. In contrast, in the trench warfare hypothesis, the evolution of both host *R* genes and pathogen *AVR* genes is nondirectional.

Rice blast is one of the most destructive diseases in rice-growing regions and is caused by the filamentous ascomycetous fungus *Magnaporthe oryzae* (synonym of *Pyricularia oryzae*). Employing resistant rice varieties with major resistance (*R*) genes is considered the most important strategy for controlling this disease and crop loss that is also environmentally friendly and economical. To date, ≤26 *R* genes in rice have been cloned: *Pb1*, *Pia*, *Pib*, *Pid2*, *Pid3*, *Pik*, *Pikh/Pi54*, *Pikm*, *Pikp*, *Pish*, *Pit*, *Pita*, *Pizt*, *Pi1*, *Pi2*, *Pi5*, *Pi9*, *pi21*, *Pi25*, *Pi36*, *Pi37*, *Pi56*, *Pi63*, *PiCO39* (http://www.ricedata.cn/gene/gene_pi.htm), *Pi64* [2] and *Pigm* [3].

Rice resistance genes can recognize the corresponding *AVR* of *M. oryzae* and initiate their immune reaction. To date, 12 *AVR* genes in *M. oryzae* have been cloned: *AVR-Pi54* [4], *AVR-Pi9* [5], *AVR-Pib* [6], *AVR-Pia* [7], *AVR-Pii* [7], *AVR-Pik/km/kp* [7], *AVR-Pizt* [8], *ACE1* [9], *AVR-Pita* [10], *AVR1-CO39* [11], *PWL1* [12], and *PWL2* [13]. The *AVR-Pik/km/kp* gene of *M. oryzae* determines the effectiveness of the *R* gene *Pik/km/kp*. *AVR-Pik/km/kp* encodes a putative secreted protein with 113 amino acids and two conserved motifs: motif-1, [LI]xAR[SE][DSE], and motif-2, [RK]CxxCxxxxxxxxxxxxH (similar to the C2H2 zinc finger motif) [7]. The *AVR-Pik/km/kp* gene was cloned from an isolate of Ina168 but found to be absent in the assembled sequence of isolate 70–15, which is recognized by the host Pik resistance protein and triggers the defense response [7]. Five *AVR-Pik* alleles (*AVR-Pik-A*, *AVR-Pik-B*, *AVR-Pik-C*, *AVR-Pik-D*, and *AVR-Pik-E*) were found [7], and *AVR-Pik-D* (20.5%) and *AVR-Pik-E* (1.4%) were detected among 77 isolates [14]. Four *AVR-Pik* alleles (*AVR-Pik-A*, *AVR-Pik-C*, *AVR-Pik-D*, and *AVR-Pik-E*) were found among 39 isolates worldwide (three isolates from Europe, six isolates from America, seven isolates from Africa and 23 isolates from Asia), and *AVR-Pik-D* was the most frequent allele (15 out 39), while the *AVR-Pik-A*, *AVR-Pik-C*, and *AVR-Pik-E* alleles had similar frequencies (7–9 out of 39) [15]. *AVR-Pik/km/kp* has evolved via gene gain/loss [7], while substitution mutations were observed in the coding regions of *AVR-Pik/km/kp* in *M. oryzae* populations, and 16 single nucleotide polymorphisms (SNPs) were found in regions without signal domains in Chinese rice blast isolates [16].

The *Pik* locus is located on the long arm of chromosome 11 and is known to have a resistance function [17–20]. At the *Pik* locus, five rice blast *R* genes (*Pik*, *Pik-m*, *Pik-p*, *Pik-h* and *Pik-s*) are involved, among which 4 *R* genes (*Pik*, *Pik-m*, *Pik-p* and *Pik-h*) have been isolated [18, 21–24] and *Pik* is regarded as the youngest allele [22]. *Pik*, *Pik-m*, *Pik-p* and *Pik-h* were cloned and found to encode a putative CC-NBS-LRR protein [18, 23, 25, 26]. The CC domain of *Pik-1* physically binds to the *AVR-Pik* effector of *M. oryzae* to trigger *Pik*-specific resistance [15, 23]. The rice resistance gene *Pik-s* is still not cloned. Monogenic lines containing 24 rice blast resistance genes, including *Pik*, *Pik-m*, *Pik-p*, *Pik-h* and *Pik-s*, were developed and will be used to characterize the pathogenicity of rice blast fungus [27].

*Pikm* and *Pikp* exhibit a high level of resistance to blast fungus from Fujian Province and can be used in parents for resistance breeding in Fujian Province [28]. *Pikm*, *Piks*, and *Pikp* are moderately resistant in Sichuan and Guizhou Provinces, China [29]. *Pikm*, *Piks*, and *Pik* are moderately resistant, while *Pikh* exhibits high resistance in Guangdong Province, China [30], and 35.4% of 82 rice germplasm resources were found to carry *Pikh* by molecular analysis [31]. Eighty of 229 rice cultivars and breeding materials carry the *Pik* locus in Fujian Province, based on PCR detection [32]. Different resistance spectra of *Pik*, *Pikm*, *Pikp*, *Pikh* and *Piks* at the *Pik* locus were detected in 282 blast isolates collected from Yunnan Province, China [33]. The *R* genes of the *Pik* locus exhibit high resistance to Chinese rice blast fungus.

Further understanding the molecular evolution of the *AVR* gene has potential implications for the development of resistance breeding, the rational use of resistance genes in production, and the deployment of more effective strategies to control the disease. Regarding the long-term interactions between the pathogen and its host, the host employs resistance genes to prevent infection by the pathogen; however, the pathogen attempts to overcome them, and the coevolution of the pathogen and its host becomes discernible at the genome level [34, 35]. The pathogen utilizes mutation to adapt to novel host alleles and the environment, while its genome structure is highly variable and impacted by host selection [15, 36, 37]. Analyzing DNA sequence variation of *AVR-Pik/km/kp* alleles of *M. oryzae* in field isolates will help to understand the effectiveness and durability of the resistance gene *Pik* alleles in China.

The goal of the present study was to analyze the DNA sequence variation of *AVR-Pik/km/kp* alleles in field isolates of *M. oryzae* to understand the variation and coevolutionary mechanism of *M. oryzae AVR-Pik/km/kp* alleles and rice *Pik* alleles in Yunnan Province.

## Results

### Efficacy of *Pik* genes and detection frequency of *AVR-Pik* alleles

Based on the disease reactions, the efficacy of the *Pik* genes *Pik*, *Pikm*, *Pikp*, *Pikh* and *Piks* were examined. Some 223, 256, 154, 276 and 83 of the 366 isolates (collected from different rice growing regions of Yunnan and selected as representative isolates) tested were avirulent to the *Pik*, *Pikm*, *Pikp*, *Pikh* and *Piks* gene-containing rice monogenic lines IRBLk-K, IRBLkm-Ts, IRBLkp-K60, IRBLkh-K3 and IRBLks-F5, respectively (Table 1). The frequency of avirulence to *Pik*, *Pikm*, *Pikp*, *Pikh* and *Piks* was 60.9, 69.9, 42.1, 75.4 and 22.7%, respectively, while the remaining 143, 110, 212, 90 and 283 isolates were virulent to the corresponding *R* gene (Table 1). Of 366 isolates, *AVR-Pik/km/kp* alleles of 278 were amplified by *AVR-Pik/km/kp* (*AVR-Pik* allele)-specific primers (pex31F/pex31R) (Table 1), and the mean percentage of the *AVR-Pik/km/kp* allele was 76.0%. The highest percentage of *AVR-Pik/km/kp* was 90.3% in the *M. oryzae* population collected from northeastern Yunnan, whereas the lowest percentage was 66.7% from northwestern Yunnan (Table 1). The percentages of *AVR-Pik/km/kp* were 77.8, 90.3, 66.7, 72.7, 89.3 and 68.3% in central, northeastern, northwestern, southeastern, southwestern and western Yunnan, respectively. Similarly, the percentages of *AVR-Pik/km/kp* were 74.5 and 77.0% in *Xian*/*Indica* (*XI*) and *Geng*/*Japonica* (*GJ*) rice-growing regions in Yunnan. These findings suggest that *Pik* loci have different effective uses in preventing blast infections in most rice production areas in Yunnan.

**Table 1:** Distribution of *AVR-Pik* genes and avirulent isolates of *M. oryzae* collected from Yunnan, China, in IRBLk-K, IRBLkm-Ts, IRBLkp-K60, and IRBLkh-K3

Locations	No. of isolates	PCR detection		Pathogenicity assay^a^				
		No. of isolates with *AVR-Pik*	Frequency (%)	No. of avirulent isolates and frequency (%)				
				IRBLk-K	IRBLkm-Ts	IRBLkp-K60	IRBLkh-K3	IRBLks-F5
Central	54	42	77.8	40 (74.1)	39 (72.2)	36 (66.7)	43 (79.6)	15 (27.8)
Northeastern	72	65	90.3	62 (86.1)	64 (88.9)	52 (72.2)	68 (94.4)	15 (20.8)
Northwestern	15	10	66.7	2 (13.3)	4 (26.7)	2 (13.3)	5 (33.3)	1 (6.7)
Southeastern	33	24	72.7	24 (72.7)	26 (78.8)	19 (57.6)	27 (81.8)	2 (6.1)
Southwestern	28	25	89.3	16 (57.1)	20 (71.4)	15 (53.6)	22 (78.6)	6 (21.4)
Western	164	112	68.3	79 (48.2)	103 (62.8)	30 (18.3)	111 (67.7)	44 (26.8)
Total	366	278	76.0	223 (60.9)	256 (69.9)	154 (42.1)	276 (75.4)	83 (22.7)
*XI*	149	111	74.5	109 (73.2)	123 (82.6)	73 (49.0)	130 (87.2)	40 (26.8)
*GJ*	217	167	77.0	114 (52.5)	133 (61.3)	81 (37.3)	146 (67.3)	43 (19.8)
Total	366	278	76.0	223 (60.9)	256 (69.9)	154 (42.1)	276 (75.4)	83 (22.7)

^a^Indicates the pathogenicity assay of the monogenic lines IRBLk-K, IRBLkm-Ts, IRBLkp-K60, IRBLkh-K3 and IRBLks-F5 containing *Pik*, *Pikm*, *Pikp*, *Pikh* and *Piks*, respectively. *XI* and *GJ* indicate *Xian*/*Indica* and *Geng*/*Japonica*, respectively

### A novel *AVR-Pikh* gene was identified to be associated with *AVR-Pik/km/kp* alleles

The *AVR-Pik/km/kp* gene is an effector gene with 342 nucleotides encoding a putative secreted protein possessing one signal peptide of 57 nucleotides in the first exon in the open reading frame (ORF) [7]. A total of 10 *AVR-Pik* haplotypes, including the five original *AVR-Pik* alleles *AVR-Pik_D* (GenBank Accession No. AB498875) (H01), *AVR-Pik_A* (AB498876) (H02), *AVR-Pik_B* (AB498877) (H03), *AVR-Pik_C* (AB498878) (H04), and *AVR-Pik_E* (AB498879) (H05), were identified based on the DNA sequence assemblies of 201 isolates (Table 2). The remaining 77 isolates were sequenced, but they had double peaks and were removed for further analysis. Five novel *AVR-Pik/km/kp* haplotypes (H06-H10) were identified. Alignment of DNA sequence assemblies of the *AVR-Pik/km/kp* gene from 201 isolates revealed six polymorphic sites in the exon region, and none of them were in the signal peptide region (Table 2). Six sites in the exon region resulted in amino acid substitutions (Table 3). Moreover, the *AVR-Pik/km/kp* allele sequence assemblies among the 201 isolates were predicted to produce 10 functional proteins (Table 3). Among these 10 proteins, amino acid variations were predicted to occur at five positions. All variations occurred throughout the protein, except for the putative secreted proteins possessing the [RK]CxxCxxxxxxxxxxxxH] motif (Table 3; Additional file 1: Figure S1). Amino acid variations at M78K were found in six isolates, all of which were virulent in the monogenic lines IRBLk-K (with *Pik*), IRBLkm-Ts (with *Pikm*), IRBLkp-K60 (with *Pikp*), IRBLkh-K3 (with *Pikh*) and IRBLks-F5 (with *Piks*) (Table 3). This finding suggests that amino acid 78 M is critical for the avirulence function of *AVR-Pik/km/kp/kh* loci. The isolates of the H01, H07 and H09 haplotypes harbored the avirulence genes *AVR-Pik/km/kp/kh*, the isolates of H05 and H08 harbored *AVR-Pik/km/kh*, the isolates of H06 harbored *AVR-Pikm/kh*, and the isolates of H02 and H03 harbored *AVR-Pikh* because these isolates were avirulent to the corresponding *R* gene(s) (Table 3). The isolates of H04 and H10 had overcome the resistance of all *Pik* alleles at the loci (Table 3). Thus, the novel avirulence gene *AVR-Pikh* was identified, and the evolution of *AVR-Pik* alleles of *M. oryzae* was involved. The 10 haplotypes did not harbor *AVR-Piks* because the isolates were virulent to the monogenic line IRBLks-F5 (harboring *Pi-ks*) (Table 3). Some 75 isolates contained *AVR-Pik/km/kp/kh* (frequency of 36.4%), 55 isolates contained *AVR-Pik/km/kh* (frequency of 26.7%), four isolates contained *AVR-Pikm/kh* (frequency of 1.9%), and 50 isolates contained *AVR-Pikh* (frequency of 24.9%). Some 17 isolates did not contain these avirulence genes (Additional file 1: Table S1). In summary, five novel *AVR-Pik* loci were identified, and 91.5% of the total isolates contained *AVR-Pikh*, which is widely distributed in southwestern China.

**Table 2:** Haplotypes of *AVR-Pik* loci in rice blast fungus in Yunnan, China

Haplotype	No. of isolates	% of total	Variant locus^a^					
			136	139	143	200	233	234
AB498875 (*AVR-Pik_D*)			C	C	G	C	T	G
AB498876 (*AVR-Pik_A*)			A	G	A	.	.	.
AB498877(*AVR-Pik_B*)			A	G	A	.	.	A
AB498878 (*AVR-Pik_C*)			A	.	.	A	.	.
AB498879 (*AVR-Pik_E*)			A	.	.	.	.	.
H01	45	22.4	.	.	.	.	.	.
H02	46	22.9	A	G	A	.	.	.
H03	4	2	A	G	A	.	.	A
H04	11	5.5	A	.	.	A	.	.
H05	51	25.4	A	.	.	.	.	.
H06	4	2	A	.	A	.	.	.
H07	27	13.4	.	.	A	.	.	.
H08	4	2	A	.	A	A	.	.
H09	3	1.5	.	G	A	.	.	.
H10	6	3	A	G	A	.	A	.

^a^Indicates the same as AB498875 (GenBank Accession No.). AB498875, AB498876, AB498877, AB498878 and AB498879 of *AVR-Pik* were obtained from GenBank and represent the five different alleles *AVR-Pik_D*, *AVR-Pik_A*, *AVR-Pik_B*, *AVR-Pik_C*, and *AVR-Pik_E*, respectively

**Table 3:** Variation in the *AVR-Pik* loci proteins in rice blast fungus in Yunnan, China

Haplotype	Total isolates	Variant locus^a^					Disease reaction^b^					Functional allele^c^
		46	47	48	67	78	IRBLk-K	IRBLkm-Ts	IRBLkp-K60	IRBLkh-K3	IRBLks-F5	
AB498875		H	P	G	A	M						*AVR-Pik/km/kp* ^d^
AB498876		N	A	D	**.**	**.**						-^d^
AB498877		N	A	D	**.**	I						-^d^
AB498878		N	**.**	**.**	D	**.**						-^d^
AB498879		N	**.**	**.**	**.**	**.**						*AVR-Pik/km* ^d^
H01	45	**.**	**.**	**.**	**.**	**.**	37R + 8 M	39R + 6 M	26R + 19 M	41R + 4 M	34S + 11 M	*AVR-Pik/km/kp/kh*
H02	46	N	A	D	**.**	**.**	35S + 11 M	27S + 19 M	38S + 8 M	44R + 2 M	45S + 1 M	*AVR-Pikh*
H03	4	N	A	D	**.**	I	4S	4S	4S	3R + 1 M	3S + 1 M	*AVR-Pikh*
H04	11	N	**.**	**.**	D	**.**	8S + 3 M	5S + 6 M	8S + 3 M	5S + 6 M	8S + 3 M	*–*
H05	51	N	**.**	**.**	**.**	**.**	28R + 23 M	49R + 2 M	49S + 2 M	49R + 2 M	26S + 25 M	*AVR-Pik/km/kh*
H06	4	N	**.**	D	**.**	**.**	3S + 1 M	3R + 1 M	4S	3R + 1 M	4S	*AVR-Pikm/kh*
H07	27	**.**	**.**	D	**.**	**.**	25R + 2 M	25R + 2 M	25R + 2 M	24R + 3 M	20S + 7 M	*AVR-Pik/km/kp/kh*
H08	4	N	**.**	D	D	**.**	4R	4R	3S + 1 M	4R	4S	*AVR-Pik/km/kh*
H09	3	**.**	A	D	**.**	**.**	3R	3R	2R + 1 M	3R	1R + 2S	*AVR-Pik/km/kp/kh*
H10	6	N	A	D	**.**	K	6S	5S + 1 M	5S + 1 M	6S	6S	–

^a^Indicates the same as AB498875

^b^Indicates the pathogenicity assay of the monogenic lines IRBLk-K, IRBLkm-Ts, IRBLkp-K60, IRBLkh-K3, and IRBLks-F5 containing the resistance genes *Pik*, *Pikm*, *Pikp*, *Pikh*, and *Piks*, respectively. R, M and S indicate that the disease reaction was resistant, moderately resistant and susceptible, respectively. (Ex.45R indicates that 45 isolates were avirulent to the corresponding monogenic line)

^c^Indicates a lack of avirulent functional alleles to the corresponding *R* genes

^d^The functional alleles from the references of Yoshida et al. [7]: AB498875, AB498876, AB498877, AB498878 and AB498879 are *AVR-Pik-D*, *AVR-Pik-A*, *AVR-Pik-B*, *AVR-Pik-C*, and *AVR-Pik-E*, respectively

### Stepwise evolution and haplotype diversity of *AVR-Pik* loci in *M. oryzae*

Among the 10 *AVR-Pik* haplotypes, the haplotypes H01, H02, H03, H04 and H05 were identical to the original *AVR-Pik* alleles of *AVR-Pik_D* (GenBank Accession No. AB498875), *AVR-Pik_A* (AB498876), *AVR-Pik_B* (AB498877), *AVR-Pik_C* (AB498878), and *AVR-Pik_E* (AB498879) (Table 2), respectively. Seven haplotypes were detected in 88, 37 and 39 *M. oryzae* isolates from western, central and northeastern Yunnan, respectively. Six haplotypes were detected in 17 *M. oryzae* isolates from southeastern Yunnan, three haplotypes were detected in 10 *M. oryzae* isolates from southwestern Yunnan, and only one haplotype was detected in 10 *M. oryzae* isolates from northwestern Yunnan (Table 4). Ten and eight haplotypes were found in the *GJ* and *XI* rice-growing regions, and the diversity index (DI) was 0.79 and 0.75 for these regions, respectively. Similarly, the DI was 0.78, 0.68, 0.65, 0.62, 0.54, and 0 for northeastern, central, western, southeastern, southwestern, and northwestern Yunnan, respectively (Table 4). In summary, the DI of *AVR-Pik* alleles was ordered in Yunnan Province as follows: northeastern>central>western>southeastern>southwestern>northwestern. The DI of *AVR-Pik* alleles in the *GJ* rice-growing region was similar to that in the *XI* rice-growing region.

**Table 4:** Distribution of *AVR-Pik* haplotypes in different rice-growing regions

Haplotype	No. isolates	Percent (%)	Regions						Production	
			Northeastern	Central	Southeastern	Western	Northwestern	Southwestern	*XI*	*GJ*
H01	45	21.8	12 (30.8)^a^	14 (37.8)	10 (58.8)	9 (10.2)	0	0	19 (30.6)	26 (18.7)
H02	46	22.3	4 (10.3)	2 (5.4)	1 (5.9)	29 (33.0)	10 (100)	0	2 (3.2)	44 (31.7)
H03	4	1.9	2 (5.1)	2 (5.4)	0	0	0	0	0	4 (2.9)
H04	11	5.3	1 (2.6)	0	2 (11.8)	2 (2.3)	0	6 (60.0)	9 (14.5)	2 (1.4)
H05	51	24.8	9 (23.1)	0	0	42 (47.7)	0	0	22 (35.5)	29 (20.9)
H06	4	1.9	0	1 (2.7)	0	0	0	3 (30.0)	3 (4.8)	1 (0.7)
H07	27	13.1	9 (23.1)	15 (40.5)	1 (5.9)	2 (2.3)	0	0	3 (4.8)	24 (17.3)
H08	4	1.9	2 (5.1)	1 (2.7)	1 (5.9)	0	0	0	1 (1.6)	3 (2.2)
H09	3	1.5	0	2 (5.4)	0	1 (1.1)	0	0	0	3 (2.2)
H10	6	2.9	0	0	2 (11.8)	3 (3.4)	0	1 (10.0)	3 (4.8)	3 (2.2)
Total	201	100	39	37	17	88	10	10	62	139
No. of haplotypes			7	7	6	7	1	3	8	10
Index of diversity^b^			0.78	0.68	0.62	0.65	0.00	0.54	0.75	0.79

^a^Number and frequency (in brackets) of isolates of each haplotype

^b^The diversity index was calculated as the frequency of haplotypes in the *M. oryzae* population following Fontaine’s method [38]: diversity index = (1-∑^n^_i = 1_p_i_^2^) (where pi is the frequency of haplotype i in a population)

**Fig. 1:**
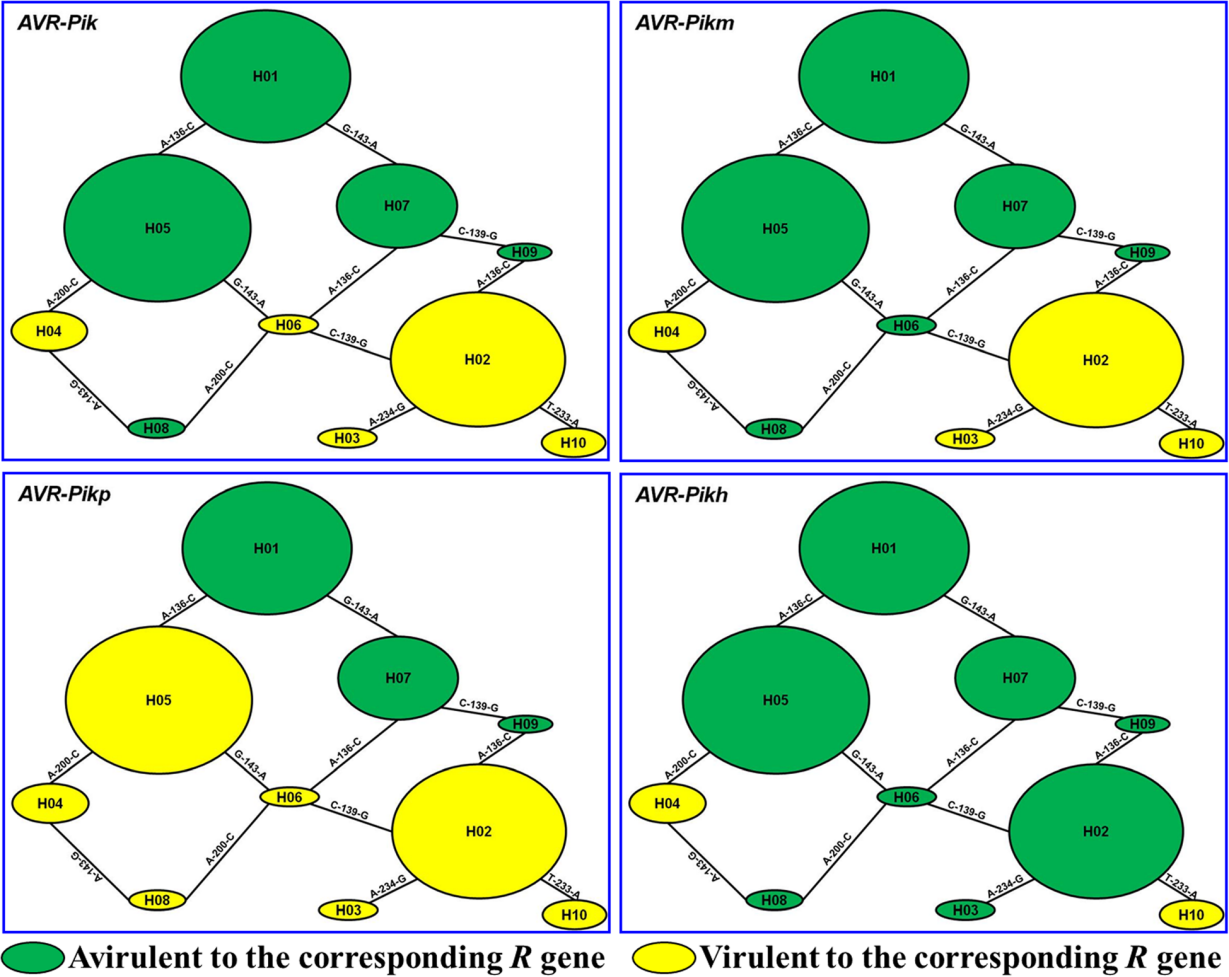
The haplotype network for the 10 *AVR-Pik* alleles. The original *AVR-Pik* allele is designated as the H01 haplotype in the network. Haplotypes are separated by mutational events. All haplotypes are displayed as circles. The size of the circles corresponds to the haplotype frequency. Haplotypes H01 to H05 are the same as AB498875, AB498876, AB498877, AB498878 and AB498879 (GenBank Accession No.) of *AVR-Pik* and were obtained from GenBank. Green indicates avirulence to the corresponding *R* gene, and yellow indicates virulence to the corresponding *R* gene

Six nucleotide variations in the exons of *AVR-Pik* alleles were observed (Additional file 1: Figure S1 and Table S2), and a haplotype network based on sequence variation was developed (Fig. 1). Four microevolutionary clusters of *AVR-Pik*, *AVR-Pikm*, *AVR-Pikp*, and *AVR-Pikh* were observed among 201 field isolates (Fig. 1). The five original *AVR-Pik* alleles *AVR-Pik_D* (H01), *AVR-Pik_A* (H02), *AVR-Pik_B* (H03), *AVR-Pik_C* (H04), and *AVR-Pik_E* (H05) were involved in the networks. The isolates of H01, H05, H07, H08 and H09 were avirulent to IRBLk-K (with *Pik*), whereas the isolates of H02, H03, H04, H06 and H10 were virulent to *Pik* (Table 3; Fig. 1). The isolates of H01, H05, H06, H07, H08 and H09 were avirulent to IRBLkm-Ts (with *Pikm*), whereas the isolates of H02, H03, H04, and H10 were virulent to *Pikm* (Table 3; Fig. 1). The isolates of H01, H07 and H09 were avirulent to IRBLkp-K60 (with *Pikp*), whereas the isolates of H02, H03, H04, H05, H06, H08 and H10 were virulent to *Pikp* (Table 3; Fig. 1). The isolates of H01, H02, H03, H05, H06, H07, H08 and H09 were avirulent to IRBLkh-K3 (with *Pikh*), whereas the isolates of H04 and H10 were virulent to *Pikh* (Table 3; Fig. 1). These findings suggest that there were four distinct stepwise-evolved patterns (*AVR-Pik, AVR-Pikm, AVR-Pikp,* and *AVR-Pikh*) in rice-growing regions of Yunnan.

**Fig. 2:**
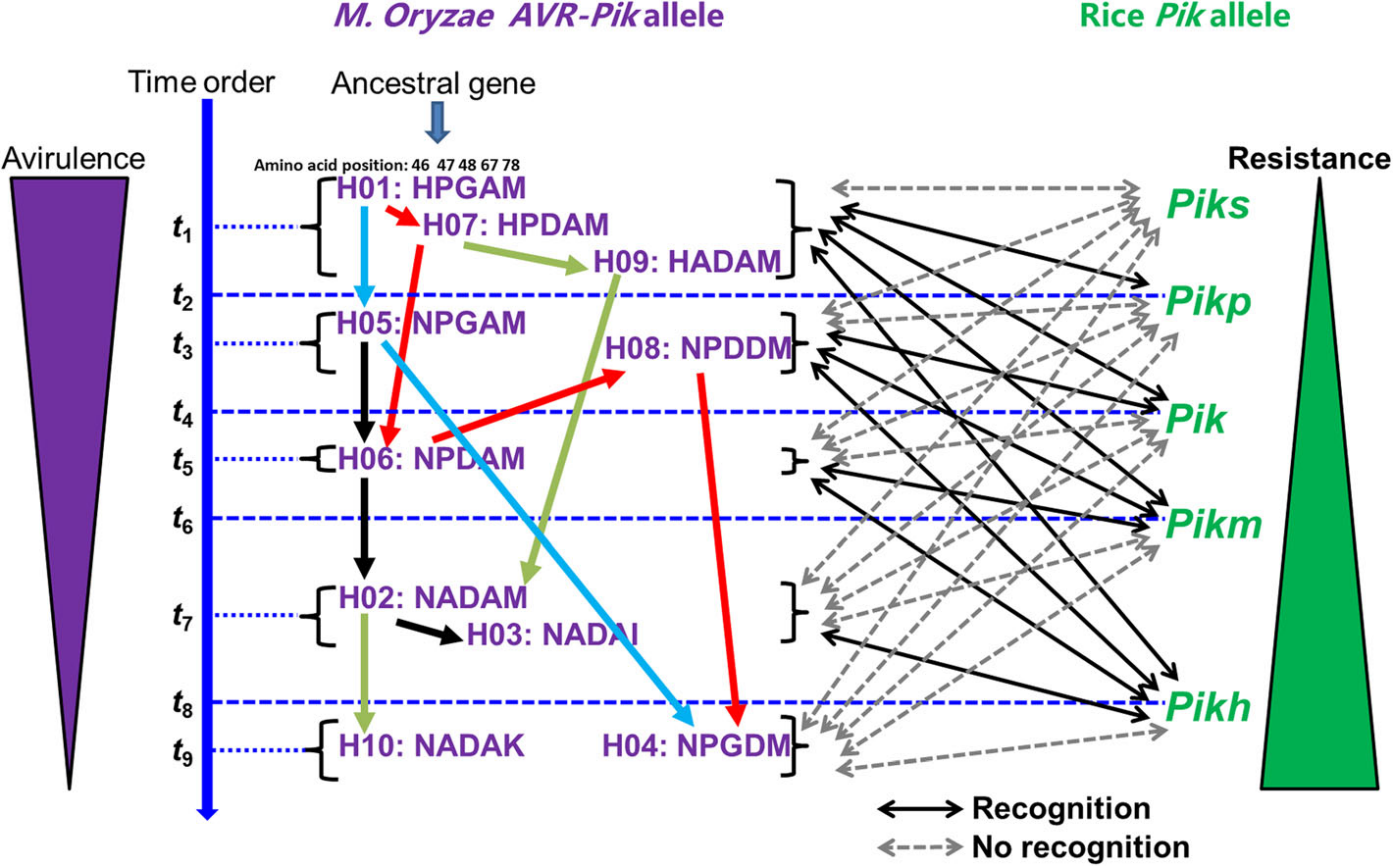
Possible scenario for *M. oryzae AVR-Pik* allele-rice *Pik* allele interactions and coevolution. Chronological order is given on the left (time order). The *AVR-Pik* homolog H01 (*AVR-Pik-D*) was derived from an ancestral *M. oryzae* gene. *AVR-Pik-D* (H01), H07 and H09 are recognized by *Pikp*; thus, the altered alleles *AVR-Pik-E* (H05) and H08 evolved. In response to this situation, another *Pik* allele, *Pik*, evolved that can recognize five alleles, namely, *AVR-Pik-D* (H01), H07, H09, *AVR-Pik-E* (H05) and H08. Then, yet another *AVR-Pik* allele, H06, was derived that cannot be recognized by *Pikp* and *Pik*. Next, the rice *R* gene *Pikm* was utilized that recognizes *AVR-Pik-D* (H01), H07, H09, *AVR-Pik-E* (H05), H08 and H06. Then, two more *AVR-Pik* alleles, namely, *AVR-Pik-A* (H02) and *AVR-Pik-B* (H03), were derived that cannot be recognized by *Pikp*, *Pik* and *Pikm*. Next, the rice *R* gene *Pikh* was utilized that recognizes *AVR-Pik-D* (H01), H07, H09, *AVR-Pik-E* (H05), H08, H06, *AVR-Pik-A* (H02) and *AVR-Pik-B* (H03). Then, two other *AVR-Pik* alleles, namely, *AVR-Pik-C* (H04) and H10, evolved that cannot be recognized by any of the five *Pik* alleles

A possible scenario for *M. oryzae AVR-Pik* allele-rice *Pik* allele interactions and coevolution was constructed (Fig. 2). The *AVR-Pik* homolog H01 (*AVR-Pik-D*) was derived from an ancestral *M. oryzae* gene. The *Pik* allele, *Piks*, cannot recognize the three alleles *AVR-Pik-D* (H01), H07 and H09; thus, the other *Pik* allele, *Pikp*, evolved that can recognize these three alleles, while the altered alleles H05 (*AVR-Pik-E*) and H08 evolved to virulence from avirulence via nucleotide substitution to avoid recognition by *Pikp* (Table 2; Fig. 2). For this situation, another *Pik* allele, *Pik*, evolved that can recognize five alleles, namely, *AVR-Pik-D* (H01), H07, H09, *AVR-Pik-E* (H05) and H08. Then, yet another *AVR-Pik* allele, H06, was derived that cannot be recognized by *Pikp* and *Pik*. Next, the rice *R* gene *Pikm* was utilized that recognizes *AVR-Pik-D* (H01), H07, H09, *AVR-Pik-E* (H05), H08 and H06. Then, two more *AVR-Pik* alleles, namely, *AVR-Pik-A* (H02) and *AVR-Pik-B* (H03), were derived that cannot be recognized by *Pikp*, *Pik* and *Pikm*. Next, the rice *R* gene *Pikh* was utilized that recognizes *AVR-Pik-D* (H01), H07, H09, *AVR-Pik-E* (H05), H08, H06, *AVR-Pik-A* (H02) and *AVR-Pik-B* (H03). Then, another two *AVR-Pik* alleles, namely, *AVR-Pik-C* (H04) and H10, evolved that cannot be recognized by any of the five *Pik* alleles (Table 2; Fig. 2). These patterns show the stepwise evolution of *AVR-Pik* and *Pik* interaction and coevolution. Interestingly, the *AVR-Pik* allele H07 was derived from H01, which can be recognized by *Pikp*, *Pik* and *Pikm*. Thus, the altered allele H06 from H07 can avoid recognition by *Pikp* and *Pik*; next, the altered allele H08 from H06 can avoid recognition by *Pikp*, while the altered allele H04 from H08 avoids recognition by any of the five *Pik* alleles. Similarly, the H09 allele was derived from H07, which can be recognized by *Pikp*, *Pik*, *Pikm* and *Pikh*; thus, the altered allele H02 allele from H09 can avoid recognition by *Pikp*, *Pik*, and *Pikm* (Table 2; Fig. 2). The H05 allele can be recognized by *Pik*, *Pikm* and *Pikh*, while the altered allele H04 from H05 can avoid recognition by *Pikp*, *Pik*, and *Pikm* (Table 2; Fig. 2). These results suggest that the avoidance evolution of *AVR-Pik* loci of *M. oryzae* was involved in the interaction and coevolution with the *Pik* loci of *M. oryzae* in nature.

### Selection pressure on *AVR-Pik* in *M. oryzae*

**Fig. 3:**
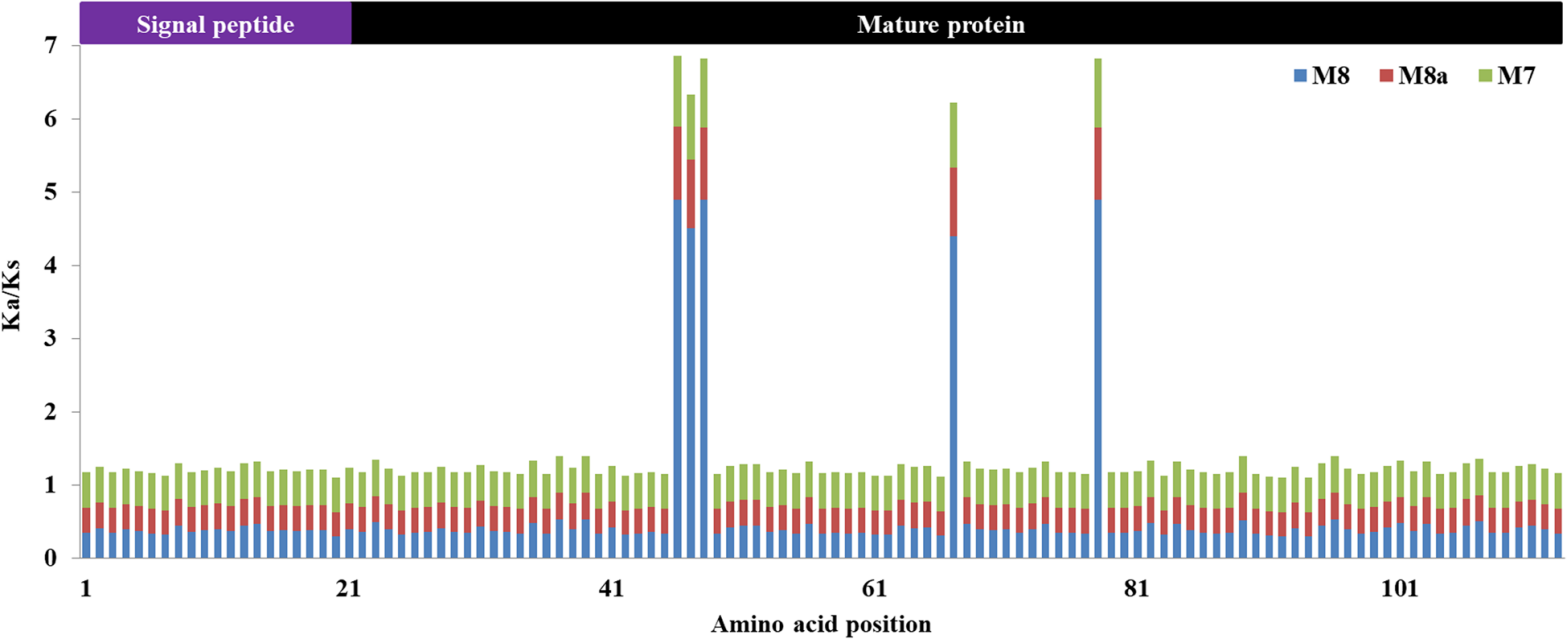
Sliding window of positively selected sites in the AVR-Pik alleles under the M8, M8a, and M7 models. The Y-axis indicates the ratio of the rate of nonsynonymous substitutions (Ka) to the rate of synonymous substitutions (Ks) (Ka/Ks); the X-axis indicates the position of the AVR-Pik amino acids in the site. The signal region of the variant structure is purple, and the black area represents the mature protein region on the label at the top of the figure

To determine the natural selection pressure on *AVR-Pik* in *M. oryzae* in Yunnan, Tajima’s neutrality of *AVR-Pik* in *M. oryzae* was tested based on 201 *AVR-Pik* DNA sequences, and Tajima’s *D* was found to be 1.19854 (Additional file 1: Table S2). The result suggests that *AVR-Pik* might be under strong population expansion or either in positive selection. The results of three positive-selection models were highly consistent (Fig. 3). The sliding window shows the distribution of the Ka/Ks values across all 113 amino acids under the M8, M8a, and M7 models (Fig. 3). The results show that the Ka/Ks value of the 46th, 47th, 48th, 67th and 78th sites was > 1, suggesting that these sites were potentially subjected to purifying selection. Positively selected sites were observed only in the mature protein region among the 201 *M. oryzae* isolates with *AVR-Pik* (Fig. 3). These results showed that the amino acid sequence was conserved in the signal peptide compared with the divergent mature protein region of *AVR-Pik* in *M. oryzae*.

To confirm the resistance of *Pik* alleles in the field, we assayed seedling and panicle blast disease with monogenic lines carrying *Pik*, *Pikm*, *Pikp*, and *Pikh*, which were developed by the Japan International Research Center for Agricultural Sciences (JIRCAS) and International Rice Research Institute (IRRI) in fields in Mangshi, Lufeng and Yiliang Counties in 2015 (Additional file 1: Table S3). The result suggests that IRBLkm-Ts (with *Pikm*), IRBLkp-K60 (with *Pikp*), and IRBLkh-K3 (with *Pikh*) were resistant, while IRBLks-F5 (with *Piks*) and IRBLk-Ka (with *Pik*) were susceptible in Mangshi County (Additional file 1: Table S3). These results suggest that *M. oryzae* isolates in the population holds *AVR-Pikm/kp/kh* genes. IRBLkh-K3 (with *Pikh*) was resistant in Lufeng and Yiliang, and the monogenic lines IRBLks-F5 (with *Piks*), IRBLk-Ka (with *Pik*), IRBLkm-Ts (with *Pikm*) and IRBLkp-K60 (with *Pikp*) were susceptible in Lufeng and Yiliang Counties, suggesting that the *M. oryzae* isolates in the population harbor *AVR-Pikh*. These results are consistent with the results of PCR detection and pathogenicity assays.

## Discussion

In this study, we found five new haplotypes in the *AVR-Pik* DNA sequences among field isolates of *M. oryzae* from various rice-producing regions in Yunnan. Numerous virulent isolates of the *Pik* gene containing rice varieties were identified in field isolates collected in Yunnan, suggesting that *Pik* was eradicated in some rice production areas due to the extensive development of *Pik* in China. The *Pik* alleles have been deployed and display high rice blast resistance in China [20, 22, 32, 39]. Complete deletions have occurred in *AVR-Pik* sequences among field isolates of *M. oryzae* from various rice-producing countries [15, 16], which agrees with our results. Numerous isolates inspected from commercial rice fields containing *AVR-Pik* suggest that *Pik* has been effective in preventing rice blast disease. In Yunnan, rice cultivars with *Pikh*, *Pikp*, *Pikm*, *Piks*, and *Pik* were resistant to 81.7, 62.8, 51.9, 43.4 and 39.4% of isolates (282 isolates), respectively [33]. The corresponding values of 146 isolates from Guangdong Province were 88.4, 39.0, 0, 1.4 and 57.5%, respectively [40]. These results suggest that some *Pik* alleles have limited effects in these rice production areas. Continued analysis of *AVR-Pik* alleles in these isolates will help us understand the evolutionary mechanism of *AVR-Pik* and predict the stability and effectiveness of *Pik* allele-mediated resistance under natural conditions.

Effective variations in DNA sequences have been observed in the telomere regions of several *AVR* genes (*AVR-Pita1*, *AVR-Pia*, and *AVR-Pii*) [7, 41, 42]. The transposable element (TE) insertion in the last exon of the *ACE1* gene [9] and Pot3 inserted in *AVR-Pizt* and *AVR-Pita1* all resulted in new virulent alleles. Based on the DNA sequence analysis [8, 43, 44], four variations, namely, a point mutation, segmental deletion, complete absence (6.7%) and TE insertion, were found in *AVR-Pib*, all of which result in loss of the avirulence function [6]. Three distinct expression profiles were found among seven of 16 functional nucleotide polymorphisms in the *AVR-Pib* genes [6]. These findings showed that *M. oryzae* uses transposons to change the expression of *AVR* genes to overcome *R* genes. In the present study, the *AVR-Pik* gene was present in most blast populations (76.0%) in Yunnan (Table 1), which was similar to rice blast isolates in Hunan Province [45]. We found significantly more nucleotide variation in the protein-coding region of *AVR-Pik* alleles, resulting in changes in amino acids and suggesting that there is intense selection pressure on *AVR-Pik* alleles in Yunnan.

DNA sequence variation was found in exon regions of *AVR-Pik*, and a total of 10 haplotypes were identified based on the six variant nucleotides among 201 isolates collected from Yunnan (Table 2). Five novel variant amino acids of the *AVR-Pik* loci variants in the 201 isolates were identified in the present study, which leads towards finding of five new haplotypes. Based on the virulence analysis of the strains harboring this variation, haplotypes H01, H02, H05 and H07 are more frequent in the field isolates. This result suggests that the loss of these haplotypes may have a larger fitness penalty than the loss of other alleles in the *M. oryzae* population. These new alleles allowed us to construct a more holonomic network among different alleles of *AVR-Pik*, and some novel haplotypes were found. We also identified the putative secreted proteins possessing the [LI]xAR[SE][DSE] and [RK]CxxCxxxxxxxxxxxxH] motifs in 201 isolates with *AVR-Pik* alleles (Table 3), which was consistent with the results of Yoshida et al. [7]. Some 126, 59, 94 and 15 isolates are variations at the amino acid positions H46N, P47A, G48D, and A67D, respectively, and four and six isolates are variations at the amino acid positions M78I and M78K, respectively (Table 3). These results showed that the 46th, 47th, 48th, 67th and 78th amino acid positions were the most variable amino acid sites among proteins of AVR-Pik/km/kp/kh.

During the long coevolution of plants and pathogens, the pathogen *AVR* genes have been recognized by the cognate plant *R* genes and triggered effective defense responses. The divergences of the *AVR* genes of the pathogen were shaped by host *R* genes and changing environmental conditions. We observed that the DI of *AVR-Pik* was similar in the *XI* and *GJ* regions (Table 4), and variations in *AVR-Pik* were different between the *XI*- and *GJ*-growing regions (Table 4). These results suggest that adaptive variations have occurred in commercial rice fields in Yunnan.

Yunnan is one of the diversification centers of the cultivated Asian rice species *Oryza sativa*. The three wild species *O. rufipogon*, *O*. *officinalis* and *O*. *meyeriana* also exist in the area [46]. Over 5000 accessions of rice germplasms were collected from fields and preserved. Among them, 227 rice accessions were characterized by a set of differential rice blast isolates, and 38 and 25 of 227 rice accessions contained the rice blast resistance genes *Pik* and *Pikm*, respectively [46]. The observed Tajima’s *D* of 1.19854 (Additional file 1: Table S2) suggests that *AVR-Pik/km/kp/kh* loci may be under population expansion or purifying selection shaped by the cognate *Pik* loci in rice-growing regions of Yunnan. Most isolates carried *AVR-Pikh* and *Pikh*, with high resistance, in Yunnan and Guangdong Provinces. This pattern may be due to *Pikh* being a widely distributed resistance gene in rice accessions. These results agree with those of Zhai et al. [22].

*AVR-Pik* is recognized specifically by the *Pik* in rice, and AVR-Pik directly physically binds the N-terminal coiled-coil domain of Pik. These observations were confirmed by yeast two-hybrid and coimmunoprecipitation assays [15]. Four alleles of *AVR-Pik* (*AVR-Pik_D*, *AVR-Pik_E*, *AVR-Pik_A*, and *AVR-Pik_C*) in Japanese isolate populations coevolved with the rice *Pik* alleles *Pikp*, *Pik* and *Pikm* [15]. Four alleles of *AVR-Pik* in the Chinese *M. oryzae* population showed stepwise evolution with the rice *Pik* alleles *Pikp*, *Pik*, *Pikm,* and *Pkh* [16]. Highly variable *Pik* alleles were observed, and stepwise changes in both the *AVR-Pik* of *M. oryzae* and *Pik* of rice were found in the field [16]. These observations indicate that *AVR-Pik* has been strongly targeted by hosts [16]. In the present study, we found both avoidance and stepwise-evolved *AVR-Pik* allele-rice *Pik* allele interactions and coevolution (Table 3; Fig. 2), which implies the presence of a high diversity of rice varieties in Yunnan. The *AVR-Pik* alleles have been regularly under selection by antagonistic alleles in host populations. Similarly, the wheat-infecting lineages from Brazil and Bangladesh appeared to be genetically distinct and displayed reticulate evolution in population genomic analyses of transcriptomic SNPs [47].

A stepwise mutation process has been demonstrated for virulence acquisition in *Fusarium oxysporum* f. sp. *ciceris* and *Puccinia striiformis* f. sp. *tritici* [48–50]. In the present study, we found one major episode of mutation evolution of *AVR-Pik* alleles and seven minor mutation evolution patterns (Fig. 2). The alternative mutation pattern can seemingly convert from avirulence to virulence via occasional mutation and showed higher efficiency (Fig. 2). These results may be due to the strong positive selection pressure imposed by the corresponding *Pik* allele of the host and the environment. Similarly, *AVRL567 can convert from avirulent to virulent by a set of stepwise mutations leading to amino acid* substitution [51]. Stepwise evolution has been observed in *AVR-Pik* [15, 16]. The possible evolution of *AVR-Pik* found in the present study was more complex than expected in the rice-growing regions of Yunnan.

## Conclusion

We detected five novel haplotypes in the field population by using 201 isolates, constructed a complex network of *AVR-Pik* alleles, and evaluated the effectiveness of *Pik* alleles in rice production areas of Yunnan. Our findings support the premise that functional *AVR-Pik* possesses diversified sequence structures and can avoid recognition by hosts via multiple site variations. Haplotype H10 originates from the frequently distributed H2 haplotype, and H4 originated from H5 and/or H8. These haplotypes can overcome all detected *Pik* alleles to date. Although the H4 and H10 haplotypes have low frequencies, surveillance of these two alleles in field populations is crucial because of their high risk of increasing in abundance in the background of *Pik*-containing rice varieties. Management must retard selection on the allele, possibly by avoiding its proliferation in agricultural practices. The prediction of blast occurrence should be based on the frequency and distribution of the allele of multiple loci, e.g., *Pik* and *AVR-Pik*, in isolate populations under field conditions.

## Methods

### Rice cultivars, fungal isolates, culture, and pathogenicity assays

The *Pik*, *Pikm*, *Pikp*, *Pikh*, and *Piks* gene-containing rice monogenic lines IRBLk-K, IRBLkm-Ts, IRBLkp-K60, IRBLkh-K3 and IRBLks-F5, respectively, and the susceptible backcrossing parent Lijiangxintuanheigu (LTH, without *Pik*) were used for pathogenicity assays (the seeds were originally acquired from Japan International Research Center for Agricultural Sciences (JIRCAS), and the JIRCAS undertook the formal identification of the plant material. The seeds conserved in plant germplasm resources bank of Yunnan Academy of Agricultural Sciences). A total of 366 isolates were collected, single-spore purified, and examined. All isolates were stored at − 20 °C on filter paper and grown in petri dishes containing oatmeal agar for spore production at room temperature under blue and white fluorescent lighting. Disease reactions were determined using a modified standard pathogenicity assay, as described by Jia et al. [52]. Specifically, rice seedlings at the 3- to 4-leaf stage were placed in a plastic bag and spray inoculated with a spore suspension of 1–5 × 10^5^ spores/mL. After inoculation, the plastic bags were sealed to maintain a high relative humidity (90–100%) for 24 h before removing the plants from the bags. Subsequently, the plants were maintained in a greenhouse for an additional 6 days to allow the development of disease symptoms. The disease reactions were rated visually based on the number and extent of lesions on the second youngest leaf using the 0–5 disease scale. A value of 0–1 indicated resistant, 2 indicated moderately resistant, and 3–5 indicated susceptible. Five seedlings were used each time, the experiment was repeated once more, and the mean disease scores were used to determine resistance versus susceptibility.

### DNA preparation, PCR amplification, and DNA sequencing

Fungal isolates were grown in complete liquid media at 25 °C for six to 8 days to produce mycelia under dark conditions. DNA was then isolated from mycelia using the cetyl trimethylammonium bromide (CTAB) method [53]. The primers pex31F (5′-TCGCCTTCCCATTTTTA-3′) and pex31R (5′-GCCCATGCATTATCTTAT-3′) were used to amplify the *AVR-Pik* allele and for sequencing using the methods of Yoshida et al. [7]. Specifically, PCRs were performed using 2× Taq PCR MasterMix (Tiangen Biotech Co. Ltd., Beijing, China). Each PCR consisted of the following components: 25 μl of Taq PCR Master Mix (containing 25 U of Taq DNA polymerase, 10X Tiangen PCR buffer, 15 mM MgCl2, and 200 μM each dNTP), 1 μl of each 10 μM primer, 2 μl of fungal genomic DNA, and 21 μl of distilled water (provided in the Tiangen kit). Reactions were performed in a Bio-Rad Thermal Cycler (C1000, Bio-Rad Laboratories, Life Science Research, CA, USA) with the following PCR program: 1 cycle at 95 °C for 3 min for initial denaturation, followed by 29 cycles at 95 °C for 30 s, 60 °C for 30 s, and 72 °C for 30 s and a final denaturation at 72 °C for 7 min. All PCRs were repeated three times (20 μl for detection, 50 μl for sequencing). The size of the amplified fragment was estimated by DL2000 DNA Ladder (Tiangen Biotech Co. Ltd., Beijing, China). PCR products were sequenced using the same primers as mentioned above for PCR amplification. DNA was sequenced by Shanghai Life Technologies Biotechnology Co., Ltd. (Shanghai, China). The amplicon from each isolate was sequenced three times.

### Resistance evaluation of *Pik* alleles in the field

The monogenic lines IRBLk-Ka, IRBLkm-Ts, IRBLks-F5, IRBLkp-K60, and IRBLkh-K3 (carrying *Pik*, *Pikm*, *Piks*, *Pikp*, and *Pikh*, respectively) were planted in fields in Mangshi, Lufeng and Yiliang Counties in Yunnan Province in 2015. The seedlings and panicles were surveyed for blast disease, and the resistance was evaluated.

### Data analysis

DNA sequences of *AVR-Pik* were assembled by the Vector NTI V.10 software suite (Invitrogen, Carlsbad, California, USA) and aligned using DNASTAR V7.10 software (http://www.dnastar.com/). The number of DNA haplotypes and polymorphic sites (*π*) and the sliding window were calculated using DnaSP v5.10.01 software [54]. Haplotype network analysis was performed using TCS1.21 (http://darwin.uvigo.es/) [55]. The DI was calculated as the frequency of haplotypes or protein types in the rice blast fungus population following the method of Fontaine et al. [38]: DI = (1-∑^n^_i = 1_p_i_^2^), where pi is the frequency of haplotype i in a population. Tajima’s neutrality test was performed using MEGA V5.10. The analysis of positive selection was performed using the Selection Server program (http://selecton.tau.ac.il). Three models were used to identify the positively selected sites under the query of AVR-Pik: M8 (positive selection enabled, beta + w ≥ 1), M8a (beta + w = 1, null model), and M7 (beta, null model). The data were then imported into Microsoft Excel for statistical analysis and to draw the sliding window.

## Additional file

Additional file 1: **Figure S1.** Diversification of *AVR-Pik* in avirulent isolates. The distribution of variation in the *AVR-Pik* alleles was analyzed using a sliding window. The X-axis shows the distribution of variation within the entire region, including the signal peptide and exon of *AVR-Pik*. The lower pane indicates the corresponding schematic representation of the signal peptide and exon of *AVR-Pik*. Window length: 1; step size: 1. The π value corresponds to the level of variation at each site because it is the sum of pairwise differences divided by the number of pairs within the population. **Table S1.** Distribution of *AVR-Pik* loci in rice blast fungus. **Table S2.** Tajima’s neutrality test of *AVR-Pik* in *M. oryzae.* The analysis involved 201 nucleotide sequences of *AVR-Pik*. *m* indicates the number of sequences, *S* indicates the number of segregating sites, *Ps* indicates *S*/*n*, *Θ* indicates *p*_s_/a_1_, *π* indicates nucleotide diversity, and *D* is the Tajima test statistic. Tajima’s D: 1.19854, statistical significance: not significant, *P* > 0.10. **Table S3.** Summary of the disease reaction of monogenic lines with *Pik* alleles in fields. Pathogenicity assay of the monogenic lines IRBLk-K, IRBLkm-Ts, IRBLkp-K60, IRBLkh-K3, and IRBLks-F5 containing the resistance genes *Pik*, *Pikm*, *Pikp*, *Pikh*, and *Piks*, respectively. R and S indicate that the disease reaction was resistant and susceptible, respectively. (DOC 142 kb)

## Abbreviations

AVR: Avirulence gene
CTAB: Cetyl trimethylammonium bromide
DI: Diversity index
GJ: Geng/Japonica
Ka: The rate of nonsynonymous substitution
Ks: The rate of synonymous substitution
LTH: Lijiangxintuanheigu
ORF: Open reading frame
R: Resistance
TE: Transposable element
XI: Xian/Indica

## References

[1] Woolhouse M, Webster J, Domingo E, Charlesworth B, Levin B (2002). Biological and biomedical implications of the co-evolution of pathogens and their hosts. Nat Genet.

[2] Ma J, Lei C, Xu X, Hao K, Wang J, Cheng Z (2015). *Pi64*, encoding a novel CC-NBS-LRR protein, confers resistance to leaf and neck blast in rice. Mol Plant-Microbe Interact.

[3] Deng Y, Zhai K, Xie Z, Yang D, Zhu X, Liu J (2017). Epigenetic regulation of antagonistic receptors confers rice blast resistance with yield balance. Science.

[4] Ray S, Singh PK, Gupta DK, Mahato AK, Sarkar C, Rathour R (2016). Analysis of *Magnaporthe oryzae* genome reveals a fungal effector, which is able to induce resistance response in transgenic rice line containing resistance gene, *Pi54*. Front Plant Sci.

[5] Wu J, Kou Y, Bao J, Li Y, Tang M, Zhu X (2015). Comparative genomics identifies the *Magnaporthe oryzae* avirulence effector *AvrPi9* that triggers *Pi9*-mediated blast resistance in rice. New Phytol.

[6] Zhang S, Wang L, Wu W, He L, Yang X, Pan Q (2015). Function and evolution of *Magnaporthe oryzae* avirulence gene *AvrPib* responding to the rice blast resistance gene *Pib*. Sci Rep.

[7] Yoshida K, Saitoh H, Fujisawa S, Kanzaki H, Matsumura H, Yoshida K (2009). Association genetics reveals three novel avirulence genes from the rice blast fungal pathogen *Magnaporthe oryzae*. Plant Cell.

[8] Li W, Wang B, Wu J, Lu G, Hu Y, Zhang X (2009). The *Magnaporthe oryzae* avirulence gene *AVR-Pizt* encodes a predicted secreted protein that triggers the immunity in rice mediated by the blast resistance gene *Piz-t*. Mol Plant-Microbe Interact.

[9] Fudal I, Bohnert HU, Tharreau D, Lebrun MH (2005). Transposition of MINE, a composite retrotransposon, in the avirulence gene *ACE1* of the rice blast fungus *Magnaporthe grisea*. Fungal Genet Biol.

[10] Orbach MJ, Farrall L, Sweigard JA, Chumley FG, Valent B (2000). A telomeric avirulence gene determines efficacy for the rice blast resistance gene *Pi-ta*. Plant Cell.

[11] Farman ML, Leong SA (1998). Chromosome walking to the *AVR1-CO39* avirulence gene of *Magnaporthe grisea*: discrepancy between the physical and genetic maps. Genetics.

[12] Kang S, Sweigard JA, Valent B (1995). The *PWL* host specificity gene family in the blast fungus *Magnaporthe grisea*. Mol Plant-Microbe Interact.

[13] Sweigard JA (1995). Identification, cloning, and characterization of *PWL2*, a gene for host species specificity in the rice blast fungus. Plant Cell.

[14] Selisana SM, Yanoria MJ, Quime B, Chaipanya C, Lu G, Opulencia R (2017). Avirulence (*AVR*) gene-based diagnosis complements existing pathogen surveillance tools for effective deployment of resistance (*R*) genes against rice blast disease. Phytopathology.

[15] Kanzaki H, Yoshida K, Saitoh H, Fujisaki K, Hirabuchi A, Alaux L (2012). Arms race co-evolution of *Magnaporthe oryzae AVR-Pik* and rice *Pik* genes driven by their phyical interactions. Plant J.

[16] Wu W, Wang L, Zhang S, Li Z, Zhang Y, Lin F (2014). Stepwise arms race between *AvrPik* and *Pik* alleles in the rice blast pathosystem. Mol Plant-Microbe Interact.

[17] Kiyosawa S (1987). With genetic view on the mechanism of resistance and virulence. Jpn J Genet.

[18] Ashikawa I, Hayashi N, Yamane H, Kanamori H, Wu J (2008). Two adjacent nucleotide-binding site–leucine-rich repeat class genes are required to confer *Pikm*-specific rice blast resistance. Genetics.

[19] Xu X, Hayashi N, Wang C, Kato H, Fujimura T, Kawasaki S (2008). Efficient authentic fine mapping of the rice blast resistance gene *Pik-h* in the *Pik* cluster, using new *Pik-h*-differentiating isolates. Mol Breed.

[20] Wang L, Xu X, Lin F, Pan Q (2009). Characterization of rice blast resistance genes in the *Pik* cluster and fine mapping of the *Pik-p* locus. Phytopathology.

[21] Ashikawa I, Hayashi N, Abe F, Wu J, Matsumoto T (2012). Characterization of the rice blast resistance gene *Pik* cloned from Kanto51. Mol Breed.

[22] Zhai C, Lin F, Dong Z, He X, Yuan B, Zeng X (2011). The isolation and characterization of *Pik*, a rice blast resistance gene which emerged after rice domestication. New Phytol.

[23] Zhai C, Zhang Y, Yao N, Lin F, Liu Z, Dong Z (2014). Function and interaction of the coupled genes responsible for *Pik-h* encoded rice blast resistance. PLoS One.

[24] Sharma TR, Madhav MS, Singh BK, Shanker P, Jana TK, Dalal V (2005). High-resolution mapping, cloning and molecular characterization of the *Pi-k*^*h*^ gene of rice, which confers resistance to *Magnaporthe grisea*. Mol Gen Genomics.

[25] Chen J, Peng P, Tian J, He Y, Zhang L, Liu Z (2015). *Pik*, a rice blast resistance allele consisting of two adjacent NBS–LRR genes, was identified as a novel allele at the *Pik* locus. Mol Breed.

[26] Yuan B, Zhai C, Wang W, Zeng X, Xu X, Hu H (2011). The *Pik-p* resistance to *Magnaporthe oryzae* in rice is mediated by a pair of closely linked CC-NBS-LRR genes. Theor Appl Genet.

[27] Tsunematsu H, Yanoria MJT, Ebron LA, Hayashi N, Ando I, Kato H (2000). Development of monogenic lines of rice for blast resistance. Breed Sci.

[28] Du Y, Ruan H, Shi N, Gan L, Yang X, Chen F (2016). Pathogenicity analysis of *Magnaporthe grisea* against major *Pi*-genes and main rice varieties in Fujian Province. J Plant Prot.

[29] Zhang S, Zhong X, Qiao G, Shen L, Zhou T, Peng Y (2017). Difference in virulence of *Magnaporthe oryzae* from Sichuan, Chongqing and Guizhou. Southwest China J Agric Sci.

[30] Yang J, Chen S, Zeng L, Li Y, Chen Z, Zhu X (2008). Evaluation on resistance of major rice blast resistance genes to *Magnaporthe grisea* isolates collected from indica rice in Guangdong Province, China. Chin J Rice Sci.

[31] Xie Q, Guo J, Yang S, Chen Z, Cheng B, Huang Y (2015). Evaluation of blast resistance spectrum and identification of resistance genes in 82 rice germplasm resources. Guangdong Agric Sci.

[32] Chen Z, Tian D, Liang T, Chen Z, Hu C, Wang F (2016). Characterization of the genotypes at the rice blast resistance *Pik* locus in 229 rice cultivars and important breeding materials. Fujian J Agric Sci.

[33] Li J, Li C, Chen Y, Lei C, Ling Z (2005). Evaluation of twenty-two blast resistance genes in Yunnan using monogenetic rice lines. Acta Phytophylacica Sin.

[34] Raffaele S, Farrer R, Cano L, Studholme D, Maclean D, Thines M (2010). Genome evolution following host jumps in the Irish potato famine pathogen lineage. Science.

[35] Terauchi R, Yoshida K (2010). Towards population genomics of effector-effector target interactions. New Phytol.

[36] Stukenbrock E, McDonald B (2009). Population genetics of fungal and oomycete effectors involved in gene-for-gene interactions. Mol Plant-Microbe Interact.

[37] Daugherty M, Malik H (2012). Rules of engagement: molecular insights from host-virus arms races. Annu Rev Genet.

[38] Fontaine C, Lovett PN, Sanou H, Maley J, Bouvet J-M (2004). Genetic diversity of the shea tree (*Vitellaria paradoxa* C.F. Gaertn), detected by RAPD and chloroplast microsatellite markers. Heredity.

[39] Hua L, Wu J, Chen C, Wu W, He X, Lin F (2012). The isolation of *Pi1*, an allele at the *Pik* locus which confers broad spectrum resistance to rice blast. Theor Appl Genet.

[40] Zhu X, Yang Q, Yang J, Lei C, Wang J, Ling Z (2004). Differentiation ability of monogenic lines to *Magnaporthe grisea* in indica rice. Acta Phytopathologica Sin.

[41] Chuma I, Isobe C, Hotta Y, Ibaragi L, Futamata N, Kusaba M (2011). Multiple translocation of the *AVR-Pita* effector gene among chromosomes of the rice blast fungus *Magnaporthe oryzae* and related species. PLoS Pathog.

[42] Dai Y, Jia Y, Correll J, Wang X, Wang Y (2010). Diversification evolution of the avirulence gene *AVR-Pita1* in field isolates of *Magnaporthe oryzae*. Fungal Genet Biol.

[43] Kang S, Lebrun MH, Farrall L, Valent B (2001). Gain of virulence caused by insertion of a Pot3 transposon in a *Magnaporthe grisea* avirulence gene. Mol Plant-Microbe Interact.

[44] Zhou E, Jia Y, Singh P, Correll J, Lee FN (2007). Instability of the *Magnaporthe oryzae* avirulence gene *AVR-Pita* alters virulence. Fungal Genet Biol.

[45] Xing J, Jia Y, Peng Z, Shi Y, He Q, Shu F (2017). Characterization of molecular identity and pathogenicity of rice blast fungus in Hunan Province of China. Plant Dis.

[46] Li J, Jiang Z (1995). Breeding of Yunnan rice. Yunnan Rice.

[47] Islam MT, Croll D, Gladieux P, Soanes DM, Persoons A, Bhattacharjee P (2016). Emergence of wheat blast in Bangladesh was caused by a south American lineage of *Magnaporthe oryzae*. BMC Biol.

[48] Steele K, Humphreys E, Wellings C, Dickinson M (2001). Support for a stepwise mutation model for pathogen evolution in Australasian *Puccinia striiformis* f. sp. *tritici* by use of molecular markers. Plant Pathol.

[49] Hovmøller M, Justetson A (2007). Rates of evolution of avirulence phenotypes and DNA markers in a northwest European population of *Puccinia striiformis* f. sp. *tritici*. Mol Ecol.

[50] Jiménez-Gasco M, Milgroom M, Jiménez-Díaz R (2004). Stepwise evolution of races in *Fusarium oxysporum* f. sp. *ciceris* inferred from fingerprinting with repetitive DNA sequences. Phytopathology.

[51] Wang C, Guncar G, Forwood J, Teh T, Catanzariti A, Lawrence GJ (2007). Crystal structures of flax rust avirulence proteins AvrL567-a and -D reveal details of the structural basis for flax disease resistance specificity. Plant Cell.

[52] Jia Y, Valent B, Lee FN (2003). Determination of host responses to *Magnaporthe grisea* on detached rice leaves using a spot inoculation method. Plant Dis.

[53] Tai T, Tanksley SD (1990). A rapid and inexpensive method for isolation of total DNA from dehydrated plant tissue. Plant Mol Biol Report.

[54] Rozas J, Sánchez-Del BJ, Messeguer X, Rozas R (2003). DnaSP, DNA polymorphism analyses by the coalescent and other methods. Bioinformatics.

[55] Clement M, Posada D, Crandall K (2000). TCS: a computer program to estimate gene genealogies. Mol Ecol.

