# Supplementary material for: Novel haplotypes and networks of AVR-Pik alleles in Magnaporthe oryzae
Source: BMC Plant Biol. 2019 May 16;19:204. doi: 10.1186/s12870-019-1817-8 (PMC6524238; doi:10.1186/s12870-019-1817-8)
Supplement: Supplementary file 1 — Figure S1. Diversification of AVR-Pik in avirulent isolates. The distribution of variation in the AVR-Pik alleles was analyzed using a sliding window. The X-axis shows the distribution of variation within the entire region, including the signal peptide and exon of AVR-Pik. The lower pane indicates the corresponding schematic representation of the signal peptide and exon of AVR-Pik. Window length: 1; step size: 1. The π value corresponds to the level of variation at each site because it is the sum of pairwise differences divided by the number of pairs within the population. Table S1. Distribution of AVR-Pik loci in rice blast fungus. Table S2. Tajima’s neutrality test of AVR-Pik in M. oryzae. The analysis involved 201 nucleotide sequences of AVR-Pik. m indicates the number of sequences, S indicates the number of segregating sites, Ps indicates S/n, Θ indicates ps/a1, π indicates nucleotide diversity, and D is the Tajima test statistic. Tajima’s D: 1.19854, statistical significance: not significant, P > 0.10. Table S3. Summary of the disease reaction of monogenic lines with Pik alleles in fields. Pathogenicity assay of the monogenic lines IRBLk-K, IRBLkm-Ts, IRBLkp-K60, IRBLkh-K3, and IRBLks-F5 containing the resistance genes Pik, Pikm, Pikp, Pikh, and Piks, respectively. R and S indicate that the disease reaction was resistant and susceptible, respectively. (DOC 142 kb) [file 12870_2019_1817_MOESM1_ESM.doc]

**
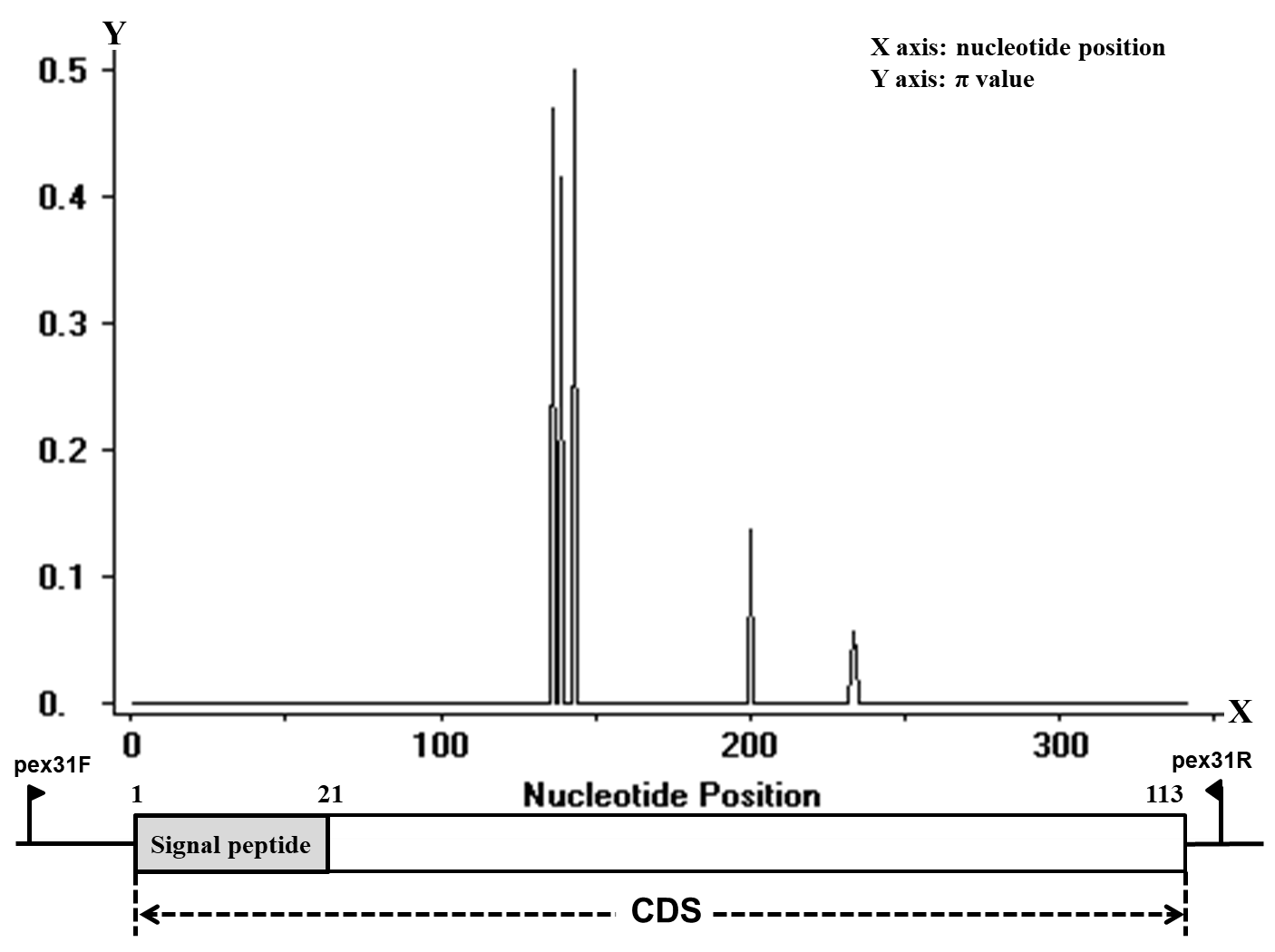
**

**Figure S1 Diversification of *AVR-Pik* in avirulent isolates.** The Distribution of variation in the *AVR-Pik* alleles was analyzed using a sliding window. The X-axis shows the distribution of variation within the entire region, including signal the peptide and exon of *AVR-Pik*. The lower pane indicates the corresponding schematic representation of the signal peptide and exon of *AVR-Pik*. Window length: 1; step size: 1. The π value corresponds to the level of variation at each site because it is the sum of pairwise differences divided by the number of pairs within the population.

**Table S1 Distribution of *AVR-Pik*** loci in rice blast fungus

|  | *AVR* gene(s) | | | | | Total |
| --- | --- | --- | --- | --- | --- | --- |
|  | *AVR-Pik/km/kp/kh* | *AVR-Pik/km/kh* | *AVR-Pikm/kh* | *AVR-Pikh* | -^a^ |  |
| Haplotype | H01, H07, H09 | H05, H08 | H06 | H02, H03 | H04, H10 |  |
| No. of isolates | 75 | 55 | 4 | 50 | 17 | 201 |
| Frequency (%) | 36.40% | 26.70% | 1.90% | 24.90% | 8.50% | 100% |

^a^ **-** Indicates the isolates of haplotypes contained without of *AVR-Pik/km/kp/kh* genes.

**Table S2 Tajima's neutrality test of *AVR-Pik* in *M*. *oryzae***a

| *m* | *S* | *Ps* | *Θ* | *π* | *D* |
| --- | --- | --- | --- | --- | --- |
| 201 | 6 | 0.017544 | 0.00298 | 0.00475 | 1.19854 (NS, P>0.10) |

^a^ The analysis involved 201 nucleotide sequences of *AVR-Pik*. *m* indicates the number of sequences, *S* indicates the number of segregating sites, *Ps* indicates *S*/*n*, *Θ* indicates *p*_s_/a_1_, *π* indicates nucleotide diversity, and *D* is the Tajima test statistic. Tajima's D: 1.19854, statistical significance: not significant, P>0.10.

**Table S3 Summary of the disease reaction of monogenic lines with *Pik* alleles in fields**

|  | County | Elevation  (m) | Eco-type | Disease reaction^a^ | | | | |
| --- | --- | --- | --- | --- | --- | --- | --- | --- |
|  |  |  |  | IRBLks-F5 | IRBLk-Ka | IRBLkm-Ts | IRBLkp-K60 | IRBLkh-K3 |
| Planting  location | Mangshi | 800 | indica | S | S | R | R | R |
|  | Lufeng | 1360 | indica | S | S | S | S | R |
|  | Yiliang | 1980 | japonica | S | S | S | S | R |

**^a^** Indicates pathogenicity assay of the monogenic lines IRBLk-K, IRBLkm-Ts, IRBLkp-K60, IRBLkh-K3, and IRBLks-F5 containing the resistanc genes *Pik*, *Pikm*, *Pikp*, *Pikh*, and *Piks*, respectively. R and S indicate that the disease reaction was resistant and susceptible, respectively.
